# Inconsistency in steroid use as antiemetics in clinical trial protocols involving immune checkpoint inhibitors combined with chemotherapy

**DOI:** 10.1002/cam4.7142

**Published:** 2024-03-28

**Authors:** Soh Mee Park, Yu Jung Kim, Ju‐Yeun Lee

**Affiliations:** ^1^ College of Pharmacy and Research Institute of Pharmaceutical Sciences Seoul National University Seoul Republic of Korea; ^2^ Department of Pharmacy Seoul National University Bundang Hospital Seongnam Republic of Korea; ^3^ Division of Hematology and Medical Oncology, Department of Internal Medicine, Seoul National University College of Medicine Seoul National University Bundang Hospital Seongnam Republic of Korea

**Keywords:** cancer management, check point control, clinical trials, immunology, medical oncology

## Abstract

**Objectives:**

This study aims to investigate the use of steroids as antiemetics in clinical trials involving immune checkpoint inhibitors with chemotherapy.

**Methods:**

Focusing on phase III trials registered before August 2023, it evaluated the consistency of steroid use guidelines.

**Results:**

Out of 3452 trials screened, 44 were selected for in‐depth review. The findings indicate a considerable variation: 13 trials did not specify the use of antiemetics, while 31 provided criteria for antiemetics, with 13 conforming to local standards, six to international guidelines, and five allowing either. Seven trials recommended effective antiemetics without detailed criteria. This inconsistency led to a range of steroid dosages, with only 11 trials advocating for minimizing or avoiding steroids for antiemetic purposes.

**Conclusion:**

The research highlights the lack of uniformity in antiemetic steroid use in trials, reflecting diverse clinical practices and underscoring the need for further research to understand the implications on treatment outcomes.

## 1 INTRODUCTION

Immune checkpoint inhibitors (ICIs) represent a major advance in cancer treatment, with significant improvements in survival across a range of cancers. These therapies work by counteracting the immunosuppressive tactics of cancer cells, enabling T cells to effectively target and destroy them.^1^

Despite their promise, the response to ICIs varies among patients, highlighting the need for research into biomarkers to predict favorable responses.^2^ Concomitant medications like steroids, which affect immune function, may influence the efficacy of ICIs,^3^ while also playing a complex role in managing cancer‐ and chemotherapy‐related symptoms.^4^

The cancer treatment landscape with ICIs is shifting from monotherapy to combination with other anticancer agents.^5^ This shift demands a deeper look into the necessary use of steroids. The use of corticosteroids, while not essential for ICI monotherapy, are often necessary in ICI‐chemotherapy combinations. For instances, taxane treatments involve two 20 mg doses for paclitaxel and three 16 mg doses for docetaxel, addressing hypersensitivity and edema.^6^ Platinum‐based chemotherapies necessitate 8–12 mg of dexamethasone over 3–4 days to prevent nausea and vomiting.^7^

Although numerous studies have explored the effect of steroids on ICI treatment outcomes,^4^, ^8^, ^9^, ^10^, ^11^ most have focused on ICI monotherapy. Steroids generally have a negative effect on the efficacy of immunotherapies, influenced by factors such as the timing and purpose of administration.^12^ Research on the use of steroids as a prophylactic measure, particularly as antiemetics, remains limited. Given their immunosuppressive nature, the potential influence of steroids on ICI effectiveness, even temporary use, needs more investigation. This study systematically reviewed antiemetic steroid use in clinical trial protocols for ICIs combined with chemotherapy, a critical step towards aligning research outcomes with clinical practice.

## 2 METHODS

This review was conducted in accordance with the Preferred Reporting Items for Systematic reviews and Meta‐Analyses (PRISMA) guidelines.^13^ It involved a comprehensive analysis of clinical trials involving ICIs in combination with parenteral anticancer agents with moderate to high emetogenic risk, specifically focusing on recommending steroid use as an antiemetic. The evaluated ICIs included programmed cell death 1 (PD‐1) or programmed cell death ligand 1 (PD‐L1) inhibitors approved by the Food and Drug Administration (FDA), such as atezolizumab, avelumab, cemiplimab, dostarlimab, durvalumab, nivolumab, and pembrolizumab. The emetogenic potential of anticancer agents was determined based on the guidelines provided by the National Comprehensive Cancer Network (NCCN).^7^

A detailed electronic search on clinicaltrials.gov used keywords like “combination,” “combinations,” “combined,” and “chemotherapy” along with the specific names of ICIs, limited to studies posted until August 31, 2023. Exclusions were made for indeterminate phase or phase 4 studies and those involving children under 17 years.

Our review process involved scrutinizing the titles and detailed descriptions of the retrieved trials. Eligible studies were defined as: (1) phase III clinical trials, (2) trials conducted with adult participants, (3) trials investigating a combination of ICIs and systemic chemotherapy, and (4) trials incorporating parenteral anticancer drugs with moderate‐to‐high emetogenic potential. Trials without accessible research protocols, either through clinicaltrials.gov or their publications, were excluded.

The analysis included reviewing cancer indications and types of anticancer agents used in the combination regimen. We carefully extracted and assessed all pertinent data pertaining to patient inclusion and exclusion criteria related to steroid use and the administration of antiemetics, including steroids. Specifically, regarding the use of antiemetic steroids, we thoroughly reviewed and categorized study protocols that provided explicit instructions for their use or placed restrictions on their use.

## 3 RESULTS

The initial database search across various ICIs resulted in 3452 trials (Figure S1). Refining to phase III trials of ICIs with systemic chemotherapy in adults reduced the trials to 296, and after removing duplicates to 276 trials. Of these, 147 trials specifically focused on combination with moderate‐to‐high emetogenic chemotherapy. Limited availability of detailed protocols led to excluding 103 trials. Consequently, 44 trials were included in the analysis.

In these 44 trials, pembrolizumab emerged as the most investigated ICI and was featured in 15 studies. Lung cancer was the predominant cancer type in 21 studies. The combination therapies employed in these trials universally involved parenteral cytotoxic anticancer agents, with 41 of 44 trials including regimens with a high emetogenic risk (Table 1).

**TABLE 1:** Clinical trial characteristics (*N* = 44).

	Number of trials *N* (%)
Immune checkpoint inhibitors	
Atezolizumab	10 (23)
Avelumab	1 (2)
Cemiplimab	2 (5)
Durvalumab	6 (14)
Nivolumab	10 (23)
Pembrolizumab	15 (34)
Cancer type	
Lung cancer	21 (48)
Upper GI cancer (esophagus and stomach)	7 (16)
Breast cancer	4 (9)
Head and neck cancer	4 (9)
Others	8 (18)
Treatment setting	
Neoadjuvant/adjuvant	7 (16)
First line treatment	35 (80)
Second line treatment	2 (5)
Combination chemotherapy	
Cytotoxic agent	44 (100)
Monoclonal antibody	6 (14)
Immune checkpoint inhibitor	4 (9)
Oral anticancer agent	1 (2)
Emetogenic risk of combination therapy	
High risk	41 (93)
Moderate risk	3 (7)

Focusing on the criteria for antiemetic medication use, we found that 13 trials (29.5%) of the 44 trials did not specify the use of antiemetics but allowed temporary use of steroids, whereas 31 trials (70.5%) delineated criteria for antiemetic steroid use (Table 2). Of the latter, 13 trials (29.5%) recommended following local standards for antiemetic use, and of these, five (11.4%) also suggested compliance with the manufacturer's instructions. Eleven studies (25.0%) adhered to international guidelines, and of these, five studies (11.4%) allowed the use of either local or international standards. These guidelines for antiemetics varied in their sources, including the Multinational Association of Supportive Care in Cancer (MASCC) in five studies, NCCN in four studies, the American Society of Clinical Oncology (ASCO) in one study, and a choice between NCCN and ASCO in another. The steroid dosages used in these guidelines are summarized in Table S1. Additionally, the remaining seven (15.9%) of 31 studies underlined the importance of effective antiemetic agents without specifying precise criteria.

**TABLE 2:** Summary of reference from 31 clinical trials proposing criteria for the antiemetic steroid use.

ICI	Clinical trial acronym	NCT number	Local standard	Manufacturer's instruction	International guidelines	Minimizing steroid
Atezolizumab	IMpower150	NCT02366143	√	√		
	IMpower130	NCT02367781	√	√		
	IMpower131	NCT02367794	√	√		
	IMpower132	NCT02657434	√	√		√
	IMpower133	NCT02763579	√	√		√
	IMvigor130	NCT02807636			√	√
	IMagyn050	NCT03038100	√			√
	IMpassion031	NCT03197935				√
	IMpassion050	NCT03726879				√
Avelumab	JAVELIN HEAD AND NECK 100	NCT02952586			√	
Cemiplimab	EMPOWER‐Lung 3	NCT03409614				
	EMPOWER‐Lung 2	NCT03515629				
Durvalumab	AEGEAN	NCT03800134			√	√
	CALLA	NCT03830866				
	TRIPLEX	NCT05223647	√			
Nivolumab	CheckMate 227	NCT02477826	√			
	CheckMate 722	NCT02864251	√			
	CheckMate 816	NCT02998528	√			
	CheckMate 648	NCT03143153	√			
	CheckMate 9LA	NCT03215706	√			
	ECHO‐309	NCT03348904	√			
	CA209‐9TM	NCT03349710				
Pembrolizumab	KEYNOTE‐048	NCT02358031				√
	KEYNOTE‐062	NCT02494583	√		√	
	KEYNOTE‐189	NCT02578680			√	
	KEYNOTE‐407	NCT02775435			√	
	KEYNOTE‐604	NCT03066778			√	
	KEYNOTE‐671	NCT03425643	√		√	
	KEYNOTE‐811	NCT03615326	√		√	√
	KEYNOTE‐859	NCT03675737	√		√	√
	NRG‐GY018	NCT03914612	√		√	√

The study protocols lacked specific information on the use and dosage of steroids for the local standards, and there were discrepancies in the criteria for antiemetics as outlined in several international guidelines. Notably, only 11 trials (25.0%) suggested either minimizing or avoiding steroids for antiemetic purposes. Detailed criteria for steroid use in each study protocol are listed in Table S2.

## 4 DISCUSSION

This investigation underscores the absence of uniform guidelines for steroid use as an antiemetic in clinical trial protocols exploring the combination of ICIs with conventional chemotherapy. A key challenge encountered was determining the precise dosage and duration of steroid administration as most trials followed individual institutional guidelines which varied considerably. This variability was further complicated by differing international guidelines for antiemetic use. For instance, although the NCCN classifies carboplatin doses of AUC 4 or higher as high risk,^7^ ASCO and MASCC consider them as moderate risk.^14^, ^15^ This disparity in emetogenic risk assessment likely leads to diverse practices in steroid usage for emesis prevention, especially as 31 of the 44 analyzed trials involved carboplatin in combination therapy.

Steroid administration in the context of ICI treatment occurs under various scenarios: chronic use for pre‐existing conditions, management of immune‐related adverse effects from ICIs, or preventive measures against chemotherapy‐induced emesis.^16^ Previous studies have shown that baseline steroid use exceeding 10 mg of prednisolone may lead to poor outcomes in ICI treatments.^17^ Our analysis indicates that clinical trial protocols consistently required discontinuing steroid use 1–2 weeks before starting the study. Interestingly, using steroids to manage serious immune‐related adverse effects does not appear to negatively affect the efficacy of ICI treatments,^4^ and clinical trial protocols universally recommend their use for severe immune‐related adverse effects.

The consistent outline in study protocols for using steroids for these purposes poses challenges in further limiting steroid use. The impact of antiemetic steroids, the primary focus of this study, remains controversial. However, few prospective studies have evaluated the effect of short‐term steroid use on the effectiveness of ICIs. A preclinical study indicates that dexamethasone can significantly reduce the antitumor response in ICI and chemotherapy combinations,^18^ with similar observations in non‐small cell lung cancer (NSCLC) patients receiving ICI monotherapy, suggesting that early steroid use may diminish the efficacy of ICIs.^19^ While a meta‐analysis highlighted the negative impact of steroids on survival, specific effects as antiemetics, dosage, and duration were not detailed,^20^ and a study in non‐squamous NSCLC showed that antiemetic steroids did not significantly impact survival, albeit with a small sample size.^21^ Given that chemotherapy is generally recommended only in the early stages of combination therapy, dexamethasone administered as a premedication for chemotherapy could potentially compromise the effectiveness of ICIs.

A recently published ASCO guideline indicated that there is insufficient clinical evidence to advise against dexamethasone in antiemetic regimens for patients undergoing ICI and chemotherapy, as studies like KEYNOTE‐189 and KEYNOTE‐407 have shown a survival benefit despite the use of potent antiemetic steroids.^14^ However, the latest NCCN guidelines allow dexamethasone doses to be tailored for antiemetic purposes and also suggest a one‐day dexamethasone‐sparing strategy for patients intolerant to steroids.^7^ Research by Tieri et al. comparing steroid‐containing and steroid‐free antiemetic regimens in chemoimmunotherapy, found no significant differences in nausea control or ICI therapy duration.^22^

Given these insights and the known immunosuppressive effects of steroids, it is prudent to limit their use in ICI‐ chemotherapy combination, possibly to a single day for moderately emetogenic chemotherapy as suggested by MASCC guideline, while awaiting further study. Further research on the impact of antiemetic steroid use on prognosis, along with establishing specific guidelines for their use in ICI combination regimens, is essential given the lack of stringent restrictions in current clinical practice compared to clinical trials.

## 5 CONCLUSION

This study underscores the inconsistencies in steroid use as an antiemetic in clinical trial protocols involving ICIs in combination with conventional chemotherapy. It emphasizes the need for contextual decision‐making regarding steroid administration for antiemetic purposes and calls for further investigation into how this practice affects treatment outcomes.

## AUTHOR CONTRIBUTIONS

**Soh Mee Park:** Conceptualization (equal); data curation (equal); investigation (equal); methodology (equal); writing – original draft (equal). **Yu Jung Kim:** Conceptualization (equal); methodology (equal); validation (equal); writing – review and editing (equal). **Ju‐Yeun Lee:** Conceptualization (equal); methodology (equal); validation (equal); writing – review and editing (equal).

## FUNDING INFORMATION

This research did not receive any specific grant from funding agencies in the public, commercial or not‐for‐profit sectors.

## CONFLICT OF INTEREST STATEMENT

The authors declare no conflicts of interest.

## Supporting information


Data S1.

## Data Availability

The datasets for this study are available from the corresponding author upon reasonable request.
