# Supplementary material for: Inconsistency in steroid use as antiemetics in clinical trial protocols involving immune checkpoint inhibitors combined with chemotherapy
Source: Cancer Med. 2024 Mar 28;13(7):e7142. doi: 10.1002/cam4.7142 (PMC10974703; doi:10.1002/cam4.7142)
Supplement: Supplementary file 1 — Data S1. [file CAM4-13-e7142-s001.docx]

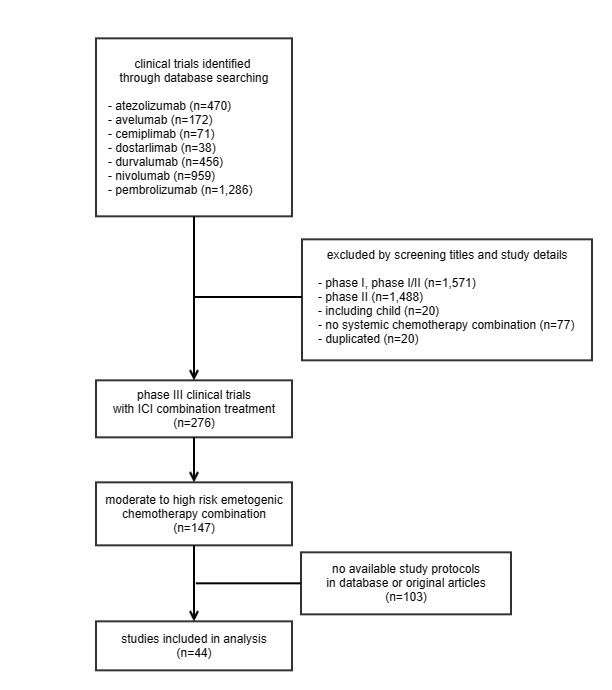


**Supplementary figure 1. Flow chart of study selection**

**Supplementary table 1. Recommended doses of dexamethasone in the latest guidelines published by ASCO^1^, MASCC/ESMO^2^ and NCCN^3^**

|  | **ASCO (2020)** | **MASCC/ESMO (2023)** | **NCCN (2023)** |
| --- | --- | --- | --- |
| **High risk: Non-AC regimen** |  |  |  |
| Acute | 20 mg  (12 mg when used with aprepitant or netupitant) | 20 mg  (12 mg when used with aprepitant or netupitant) | 12 mg |
| Delayed | 8 mg bid on days 2-4  (8 mg when used with aprepitant or netupitant) | 8 mg bid on days 2-4  (8 mg when used with aprepitant or netupitant) | 8 mg for 3 days |
| **Carboplatin ≥ AUC 4** |  |  |  |
| Acute | 20 mg  (12 mg when used with aprepitant or netupitant) | 8 mg | 12 mg |
| Delayed | No | No | 8 mg for 3 days |
| **Carboplatin < AUC 4** |  |  |  |
| Acute | 20 mg  (12 mg when used with aprepitant or netupitant) | 8 mg | 12 mg |
| Delayed | No | No | 8 mg for 2 days |
| **Moderate risk** |  |  |  |
| Acute | 8 mg | 8 mg | 12 mg |
| Delayed | No  or  8 mg on days 2-3 in patients treated with cyclophosphamide, doxorubicin, oxaliplatin, and agents known to cause delayed emesis | No | 8 mg for 2 days |

**Supplementary table 2. Antiemetic steroid use criteria in 44 clinical trial protocols**

| **Clinical trial**  **acronym** | **NCT number** | **Cancer type** | **Treatment setting** | **ICI** | **Combined chemotherapy** | **Antiemetic steroid use criteria** |
| --- | --- | --- | --- | --- | --- | --- |
| IMpower150^4^ | NCT02366143 | NSCLC | 1L | atezolizumab | bevacizumab+carboplatin+paclitaxel | Patients should receive anti-emetics according to the local standard of care and manufacturer’s instruction. |
| IMpower130^5^ | NCT02367781 | NSCLC | 1L | atezolizumab | carboplatin+nab-paclitaxel | Patients should receive anti-emetics according to the local standard of care and manufacturer’s instruction. |
| IMpower131^6^ | NCT02367794 | NSCLC | 1L | atezolizumab | carboplatin+nab-paclitaxel carboplatin+paclitaxel | Patients should receive anti-emetics according to the local standard of care and manufacturer’s instruction. |
| IMpower132^7^ | NCT02657434 | NSCLC | 1L | atezolizumab | cisplatin+pemetrexed carboplatin+pemetrexed | Patients should receive anti-emetics according to the local standard of care and manufacturer’s instruction. However, premedication with steroids should be limited when clinically feasible. |
| IMpower133^8^ | NCT02763579 | SCLC | 1L | atezolizumab | carboplatin+etoposide | Patients should receive anti-emetics according to the local standard of care and manufacturer’s instruction, but minimized to the extent that is clinically feasible. |
| IMvigor130^9^ | NCT02807636 | Urothelial carcinoma | 1L | atezolizumab | carboplatin+gemcitabine cisplatin+gemcitabine | The use of a nonsteroidal anti-emetic regimen consisting of 5-HT3 receptor and NK1R antagonists is encouraged if feasible, according to NCCN guidelines. |
| ATALANTE^10^ | NCT02891824 | Ovarian cancer | 2L | atezolizumab | bevacizumab+carboplatin+paclitaxel bevacizumab+carboplatin+gemcitabine bevacizumab+carboplatin+pegylated liposomal doxorubicin | Not mentioned |
| IMagyn050^11^ | NCT03038100 | Ovarian cancer, fallopian tube cancer, peritoneal cancer | adj.  /neoadj. | atezolizumab | carboplatin+paclitaxel | Appropriate anti-emetic nonsteroidal medication should be administered prior to the initiation of chemotherapy in accordance with the local practice and standard of care. |
| IMpassion031^12^ | NCT03197935 | TNBC | Neoadj. | atezolizumab | nab-paclitaxel → cyclophosphamide+doxorubicin | Chemotherapy-induced nausea and vomiting prophylaxis and treatment should be administered as clinically indicated. Because systemic corticosteroids may attenuate the potential beneficial immunologic effects of treatment with atezolizumab, alternative agents should be considered when clinically feasible. |
| IMpassion050^13^ | NCT03726879 | Breast cancer | Neoadj. | atezolizumab | cyclophosphamide+doxorubicin  → paclitaxel+trastuzumab+pertuzumab | Chemotherapy-induced nausea and vomiting prophylaxis and treatment should be administered as clinically indicated. Because systemic corticosteroids may attenuate the potential beneficial immunologic effects of treatment with atezolizumab, alternative agents should be considered when clinically feasible. |
| JAVELIN HEAD AND NECK 100^14^ | NCT02952586 | Head and neck cancer | 1L | avelumab | cisplatin (CCRT) | Institutional or ASCO guidelines for highly emetogenic regimens should be followed. |
| EMPOWER-LUNG3^15^ | NCT03409614 | NSCLC | 1L | cemiplimab | carboplatin+gemcitabine cisplatin+gemcitabine carboplatin+paclitaxel cisplatin+paclitaxel carboplatin+pemetrexed cisplatin+pemetrexed | Patients receiving cisplatin should receive highly effective combination antiemetic therapy. |
| EMPOWER-LUNG2 | NCT03515629 | NSCLC | 1L | cemiplimab | ipilimumab+carboplatin+paclitaxel ipilimumab+cisplatin+paclitaxel ipilimumab+carboplatin+pemetrexed ipilimumab+cisplatin+pemetrexed | Patients receiving cisplatin should receive highly effective combination antiemetic therapy. |
| CASPIAN^16^ | NCT03043872 | SCLC | 1L | durvalumab | carboplatin+etoposide cisplatin+etoposide | Not mentioned |
| POSEIDON^17^ | NCT03164616 | NSCLC | 1L | durvalumab | tremelimumab+carboplatin+gemcitabine tremelimumab+cisplatin+gemcitabine tremelimumab+carboplatin+pemetrexed tremelimumab+cisplatin+pemetrexed tremelimumab+carboplatin+nab-paclitaxel | Not mentioned |
| AEGEAN^18^ | NCT03800134 | NSCLC | neoadj. | durvalumab | carboplatin+paclitaxel cisplatin+gemcitabine carboplatin+pemetrexed cisplatin+pemetrexed | Alternative anti-emetic pre-medication should be prioritized in place of steroids. Where steroid pre-medication is utilized, steroid doses should be administered in line with 2016 MASCC/ESMO guideline. |
| CALLA^19^ | NCT03830866 | Cervical cancer | 1L | durvalumab | cisplatin(CCRT) carboplatin(CCRT) | Antiemetics are recommended. Standard antiemetic regimens consist of a steroid and 5HT-antagonist, with or without a substance P antagonist. |
| TOPAZ-1^20^ | NCT03875235 | Bile duct cancer, gallbladder cancer | 1L | durvalumab | cisplatin+gemcitabine | Not mentioned |
| TRIPLEX | NCT05223647 | SCLC | 1L | durvalumab | carboplatin+etoposide(CCRT) | Suggested antiemetic medications are Day 1 ondansetron and Day 1-3 Dexamethasone 8mg. Other antiemetic therapy may be administered according to local routines. |
| CHECKMATE 227^21^ | NCT02477826 | NSCLC | 1L | nivolumab | carboplatin+gemcitabine cisplatin+gemcitabine carboplatin+pemetrexed cisplatin+pemetrexed | Antiemetic premedication will be administered according to local standards. Recommended antiemetic treatments are dexamethasone and a 5-HT3 receptor antagonist. |
| ATTRACTION-4^22^ | NCT02746796 | Gastric cancer, GEJ cancer | 1L | nivolumab | TS1+oxaliplatin capecitabine+oxaliplatin | Not mentioned |
| CHECKMATE 722 | NCT02864251 | NSCLC | 2L | nivolumab | carboplatin+pemetrexed cisplatin+pemetrexed | Antiemetic premedication will be administered according to local standards. Recommended antiemetic treatments are dexamethasone and a 5-HT3 receptor antagonist. |
| CHECKMATE 649^23^ | NCT02872116 | Gastric cancer, GEJ cancer, esophageal cancer | 1L | nivolumab | 5-FU+oxaliplatin capecitabine+oxaliplatin | Not mentioned |
| CHECKMATE 816^24^ | NCT02998528 | NSCLC | neoadj. | nivolumab | cisplatin+pemetrexed cisplatin+gemcitabine carboplatin+paclitaxel | Antiemetic premedication will be administered according to local standards. Recommended antiemetic treatments are dexamethasone and a 5-HT3 receptor antagonist. |
| TASUKI-52^25^ | NCT03117049 | NSCLC | 1L | nivolumab | bevacizumab+carboplatin+paclitaxel | Not mentioned |
| CHECKMATE 648^26^ | NCT03143153 | Esophageal cancer | 1L | nivolumab | cisplatin+5-FU | Antiemetic premedication will be administered according to local standards. Recommended antiemetic treatments are dexamethasone and a 5-HT3 receptor antagonist. |
| CHECKMATE 9LA^27^ | NCT03215706 | NSCLC | 1L | nivolumab | ipillimumab+cisplatin+pemetrexed ipillimumab+carboplatin+pemetrexed ipillimumab+carboplatin+paclitaxel | Antiemetic premedication will be administered according to local standards. Recommended antiemetic treatments are dexamethasone and a 5-HT3 receptor antagonist. |
| ECHO-309 | NCT03348904 | NSCLC | 1L | nivolumab | epacadostat+carboplatin+gemcitabine epacadostat+cisplatin+gemcitabine epacadostat+carboplatin+paclitaxel epacadostat+carboplatin+pemetrexed epacadostat+cisplatin+pemetrexed | Antiemetic premedication will be administered according to local standards. Recommended antiemetic treatments are dexamethasone and a 5-HT3 receptor antagonist. |
| CA209-9TM | NCT03349710 | Head and neck cancer | 1L | nivolumab | cisplatin(CCRT) | It is suggested that participants receive antiemetic therapy, acute and delayed, including dexamethasone, 5-HT3 serotonin receptor antagonists. |
| KEYNOTE⁠-⁠048^28^ | NCT02358031 | Head and neck cancer | 1L | pembrolizumab | cisplatin+5-FU carboplatin+5-FU | Prevention of nausea and vomiting should be managed with aprepitant and palonosetron. Initial use of dexamethasone is limited to no more than 8 mg administered on Day 1 of a treatment cycle. |
| KEYNOTE-062^29^ | NCT02494583 | Gastric cancer, GEJ cancer | 1L | pembrolizumab | cisplatin+5-FU cisplatin+capecitabine | Prevention and/or treatment of nausea and vomiting should be managed with aprepitant and palonosetron. Additionally, use of steroids for cisplatin associated anti-emetic support is allowed and is to follow the NCCN or institutional guidelines. |
| KEYNOTE⁠-⁠189^30^ | NCT02578680 | NSCLC | 1L | pembrolizumab | carboplatin+pemetrexed cisplatin+pemetrexed | Antiemetic therapy should follow MASCC/ESMO Antiemetic guidelines. |
| KEYNOTE⁠-⁠407^31^ | NCT02775435 | NSCLC | 1L | pembrolizumab | carboplatin+nab-paclitaxel carboplatin+paclitaxel | Antiemetic therapy should follow MASCC/ESMO Antiemetic guidelines. |
| KEYNOTE⁠-⁠355^32^ | NCT02819518 | TNBC | 1L | pembrolizumab | paclitaxel nab-paclitaxel carboplatin+gemcitabine | Not mentioned |
| KEYNOTE-361^33^ | NCT02853305 | Urothelial carcinoma | 1L | pembrolizumab | carboplatin+gemcitabine  cisplatin+gemcitabine | Not mentioned |
| KEYNOTE⁠-⁠522^34^ | NCT03036488 | TNBC | neoadj. | pembrolizumab | carboplatin+paclitaxel   →cyclophosphamide+doxorubicin  cyclophosphamide+epirubicin | Not mentioned |
| KEYNOTE-604^35^ | NCT03066778 | SCLC | 1L | pembrolizumab | carboplatin+etoposide cisplatin+etoposide | Antiemetic therapy should follow MASCC/ESMO Antiemetic Guidelines. |
| KEYNOTE⁠-⁠590^36^ | NCT03189719 | GEJ cancer, esophageal cancer | 1L | pembrolizumab | cisplatin+5-FU | Not mentioned |
| KEYNOTE-671^37^ | NCT03425643 | NSCLC | neoadj. | pembrolizumab | cisplatin+pemetrexed cisplatin+gemcitabine | Antiemetic therapy should follow MASCC or appropriate local guidelines. |
| KEYNOTE⁠-⁠811^38^ | NCT03615326 | Gastric cancer, GEJ cancer | 1L | pembrolizumab | trastuzumab+cisplatin+5-FU trastuzumab+oxaliplatin+capecitabine | Prevention of nausea and vomiting should be managed with aprepitant and palonosetron. Additionally, use of steroids for cisplatin is to follow the NCCN or institutional guidelines. However, caution must be exercised to prevent the overuse of steroids. |
| KEYNOTE⁠-⁠826^39^ | NCT03635567 | Cervical cancer | 1L | pembrolizumab | carboplatin+paclitaxel+/-bevacizumab cisplatin+paclitaxel+/-bevacizumab | Not mentioned |
| KEYNOTE-859^40^ | NCT03675737 | GEJ cancer | 1L | pembrolizumab | cisplatin+5-FU oxaliplatin+capecitabine | Prevention of nausea and vomiting should be managed with aprepitant and palonosetron. Additionally, use of steroids for cisplatin is to follow the NCCN or institutional guidelines. However, caution must be exercised to prevent the overuse of steroids. |
| NRG-GY018^41^ | NCT03914612 | Endometrial cancer | 1L | pembrolizumab | carboplatin+paclitaxel | An antiemetic regimen is suggested per institutional, NCCN and/or ASCO guidelines. Use of oral steroids as an antiemetic regimen is permitted, although consideration should be given to minimize steroid use as much as possible. |
| INDUCE-4 | NCT04428333 | Hean and neck cancer | 1L | pembrolizumab | feladilimab+cisplatin+5-FU feladilimab+carboplatin+5-FU | Not mentioned |

**NSCLC**, non-small cell lung cancer; **SCLC**, small cell lung cancer; **TNBC**, triple negative breast cancer; **GEJ cancer**, gastroesophageal junction cancer; **Adj.**, adjuvant treatment; **Neoadj.**, neoadjuvant treatment; **CCRT**, concomitant chemoradiotherapy; **NCCN**, The National Comprehensive Cancer Network; **ASCO**, the American Society of Clinical Oncology; **MASCC**, the Multinational Association of Supportive Care in Cancer; **ESMO**, The European Society for Medical Oncology

**Supplementary references**

1. Hesketh PJ, Kris MG, Basch E, et al. Antiemetics: ASCO Guideline Update. *Journal of Clinical Oncology*. 2020;38(24):2782-2797.

2. Herrstedt J, Clark-Snow R, Ruhlmann C, et al. 2023 MASCC and ESMO guideline update for the prevention of chemotherapy-and radiotherapy-induced nausea and vomiting. *ESMO Open*. 2024:102195.

3. National Comprehensive Cancer Network. https://www.nccn.org/professionals/physician_gls/pdf/antiemesis.pdf. Accessed August 31, 2023.

4. Socinski MA, Jotte RM, Cappuzzo F, et al. Atezolizumab for First-Line Treatment of Metastatic Nonsquamous NSCLC. *New England Journal of Medicine*. 2018;378(24):2288-2301.

5. West H, McCleod M, Hussein M, et al. Atezolizumab in combination with carboplatin plus nab-paclitaxel chemotherapy compared with chemotherapy alone as first-line treatment for metastatic non-squamous non-small-cell lung cancer (IMpower130): a multicentre, randomised, open-label, phase 3 trial. *Lancet Oncology*. 2019;20(7):924-937.

6. Jotte R, Cappuzzo F, Vynnychenko I, et al. Atezolizumab in Combination With Carboplatin and Nab-Paclitaxel in Advanced Squamous NSCLC (IMpower131): Results From a Randomized Phase III Trial. *Journal of Thoracic Oncology*. 2020;15(8):1351-1360.

7. Nishio M, Barlesi F, West H, et al. Atezolizumab Plus Chemotherapy for First-Line Treatment of Nonsquamous NSCLC: Results From the Randomized Phase 3 IMpower132 Trial. *Journal of Thoracic Oncology*. 2021;16(4):653-664.

8. Horn L, Mansfield AS, Szczęsna A, et al. First-Line Atezolizumab plus Chemotherapy in Extensive-Stage Small-Cell Lung Cancer. *New England Journal of Medicine*. 2018;379(23):2220-2229.

9. Galsky MD, Arija JÁ A, Bamias A, et al. Atezolizumab with or without chemotherapy in metastatic urothelial cancer (IMvigor130): a multicentre, randomised, placebo-controlled phase 3 trial. *Lancet*. 2020;395(10236):1547-1557. 0

10. Kurtz JE, Pujade-Lauraine E, Oaknin A, et al. Atezolizumab Combined With Bevacizumab and Platinum-Based Therapy for Platinum-Sensitive Ovarian Cancer: Placebo-Controlled Randomized Phase III ATALANTE/ENGOT-ov29 Trial. *Journal of Clinical Oncology*. 2023;41(30):4768-4778.

11. Moore KN, Bookman M, Sehouli J, et al. Atezolizumab, Bevacizumab, and Chemotherapy for Newly Diagnosed Stage III or IV Ovarian Cancer: Placebo-Controlled Randomized Phase III Trial (IMagyn050/GOG 3015/ENGOT-OV39). *Journal of Clinical Oncology*. 2021;39(17):1842-1855.

12. Mittendorf EA, Zhang H, Barrios CH, et al. Neoadjuvant atezolizumab in combination with sequential nab-paclitaxel and anthracycline-based chemotherapy versus placebo and chemotherapy in patients with early-stage triple-negative breast cancer (IMpassion031): a randomised, double-blind, phase 3 trial. *The Lancet*. 2020;396(10257):1090-1100.

13. Huober J, Barrios CH, Niikura N, et al. Atezolizumab With Neoadjuvant Anti-Human Epidermal Growth Factor Receptor 2 Therapy and Chemotherapy in Human Epidermal Growth Factor Receptor 2-Positive Early Breast Cancer: Primary Results of the Randomized Phase III IMpassion050 Trial. *Journal of Clinical Oncology*. 2022;40(25):2946-2956.

14. Lee NY, Ferris RL, Psyrri A, et al. Avelumab plus standard-of-care chemoradiotherapy versus chemoradiotherapy alone in patients with locally advanced squamous cell carcinoma of the head and neck: a randomised, double-blind, placebo-controlled, multicentre, phase 3 trial. *The Lancet Oncology*. 2021;22(4):450-462.

15. Gogishvili M, Melkadze T, Makharadze T, et al. Cemiplimab plus chemotherapy versus chemotherapy alone in non-small cell lung cancer: a randomized, controlled, double-blind phase 3 trial. *Nature Medicine*. 2022;28(11):2374-2380.

16. Paz-Ares L, Dvorkin M, Chen Y, et al. Durvalumab plus platinum–etoposide versus platinum–etoposide in first-line treatment of extensive-stage small-cell lung cancer (CASPIAN): a randomised, controlled, open-label, phase 3 trial. *The Lancet*. 2019;394(10212):1929-1939.

17. Johnson ML, Cho BC, Luft A, et al. Durvalumab With or Without Tremelimumab in Combination With Chemotherapy as First-Line Therapy for Metastatic Non–Small-Cell Lung Cancer: The Phase III POSEIDON Study. *Journal of Clinical Oncology*. 2023;41(6):1213-1227.

18. Heymach JV, Harpole D, Mitsudomi T, et al. Perioperative Durvalumab for Resectable Non–Small-Cell Lung Cancer. *New England Journal of Medicine*. 2023;389(18):1672-1684.

19. Monk BJ, Toita T, Wu X, et al. Durvalumab versus placebo with chemoradiotherapy for locally advanced cervical cancer (CALLA): a randomised, double-blind, phase 3 trial. *The Lancet Oncology*. 2023;24(12):1334-1348.

20. Oh D-Y, He AR, Qin S, et al. Durvalumab plus Gemcitabine and Cisplatin in Advanced Biliary Tract Cancer. *NEJM Evidence*. 2022;1(8):EVIDoa2200015.

21. Hellmann MD, Paz-Ares L, Bernabe Caro R, et al. Nivolumab plus Ipilimumab in Advanced Non–Small-Cell Lung Cancer. *New England Journal of Medicine*. 2019;381(21):2020-2031.

22. Kang Y-K, Chen L-T, Ryu M-H, et al. Nivolumab plus chemotherapy versus placebo plus chemotherapy in patients with HER2-negative, untreated, unresectable advanced or recurrent gastric or gastro-oesophageal junction cancer (ATTRACTION-4): a randomised, multicentre, double-blind, placebo-controlled, phase 3 trial. *The Lancet Oncology*. 2022;23(2):234-247.

23. Janjigian YY, Shitara K, Moehler M, et al. First-line nivolumab plus chemotherapy versus chemotherapy alone for advanced gastric, gastro-oesophageal junction, and oesophageal adenocarcinoma (CheckMate 649): a randomised, open-label, phase 3 trial. *The Lancet*. 2021;398(10294):27-40.

24. Forde PM, Spicer J, Lu S, et al. Neoadjuvant Nivolumab plus Chemotherapy in Resectable Lung Cancer. *New England Journal of Medicine*. 2022;386(21):1973-1985.

25. Sugawara S, Lee JS, Kang JH, et al. Nivolumab with carboplatin, paclitaxel, and bevacizumab for first-line treatment of advanced nonsquamous non-small-cell lung cancer. *Annals of Oncology*. 2021;32(9):1137-1147.

26. Doki Y, Ajani JA, Kato K, et al. Nivolumab Combination Therapy in Advanced Esophageal Squamous-Cell Carcinoma. *New England Journal of Medicine*. 2022;386(5):449-462.

27. Paz-Ares L, Ciuleanu T-E, Cobo M, et al. First-line nivolumab plus ipilimumab combined with two cycles of chemotherapy in patients with non-small-cell lung cancer (CheckMate 9LA): an international, randomised, open-label, phase 3 trial. *The Lancet Oncology*. 2021;22(2):198-211.

28. Burtness B, Harrington KJ, Greil R, et al. Pembrolizumab alone or with chemotherapy versus cetuximab with chemotherapy for recurrent or metastatic squamous cell carcinoma of the head and neck (KEYNOTE-048): a randomised, open-label, phase 3 study. *The Lancet*. 2019;394(10212):1915-1928.

29. Shitara K, Van Cutsem E, Bang YJ, et al. Efficacy and Safety of Pembrolizumab or Pembrolizumab Plus Chemotherapy vs Chemotherapy Alone for Patients With First-line, Advanced Gastric Cancer: The KEYNOTE-062 Phase 3 Randomized Clinical Trial. *JAMA Oncology*. 2020;6(10):1571-1580.

30. Gandhi L, Rodríguez-Abreu D, Gadgeel S, et al. Pembrolizumab plus Chemotherapy in Metastatic Non–Small-Cell Lung Cancer. *New England Journal of Medicine*. 2018;378(22):2078-2092.

31. Paz-Ares L, Luft A, Vicente D, et al. Pembrolizumab plus Chemotherapy for Squamous Non–Small-Cell Lung Cancer. *New England Journal of Medicine*. 2018;379(21):2040-2051.

32. Cortes J, Rugo HS, Cescon DW, et al. Pembrolizumab plus Chemotherapy in Advanced Triple-Negative Breast Cancer. *New England Journal of Medicine*. 2022;387(3):217-226.

33. Powles T, Csőszi T, Özgüroğlu M, et al. Pembrolizumab alone or combined with chemotherapy versus chemotherapy as first-line therapy for advanced urothelial carcinoma (KEYNOTE-361): a randomised, open-label, phase 3 trial. *Lancet Oncology*. 2021;22(7):931-945.

34. Schmid P, Cortes J, Pusztai L, et al. Pembrolizumab for Early Triple-Negative Breast Cancer. *New England Journal of Medicine*. 2020;382(9):810-821.

35. Rudin CM, Awad MM, Navarro A, et al. Pembrolizumab or Placebo Plus Etoposide and Platinum as First-Line Therapy for Extensive-Stage Small-Cell Lung Cancer: Randomized, Double-Blind, Phase III KEYNOTE-604 Study. *Journal of Clinical Oncology*. 2020;38(21):2369-2379.

36. Sun JM, Shen L, Shah MA, et al. Pembrolizumab plus chemotherapy versus chemotherapy alone for first-line treatment of advanced oesophageal cancer (KEYNOTE-590): a randomised, placebo-controlled, phase 3 study. *Lancet*. 2021;398(10302):759-771.

37. Wakelee H, Liberman M, Kato T, et al. Perioperative Pembrolizumab for Early-Stage Non–Small-Cell Lung Cancer. *New England Journal of Medicine*. 2023;389(6):491-503.

38. Janjigian YY, Kawazoe A, Yañez P, et al. The KEYNOTE-811 trial of dual PD-1 and HER2 blockade in HER2-positive gastric cancer. *Nature*. 2021;600(7890):727-730.

39. Colombo N, Dubot C, Lorusso D, et al. Pembrolizumab for Persistent, Recurrent, or Metastatic Cervical Cancer. *New England Journal of Medicine*. 2021;385(20):1856-1867.

40. Tabernero J, Bang YJ, Van Cutsem E, et al. KEYNOTE-859: a Phase III study of pembrolizumab plus chemotherapy in gastric/gastroesophageal junction adenocarcinoma. *Future Oncology*. 2021;17(22):2847-2855.

41. Eskander RN, Sill MW, Beffa L, et al. Pembrolizumab plus Chemotherapy in Advanced Endometrial Cancer. *New England Journal of Medicine*. 2023;388(23):2159-2170.
